# Supplementary material for: Patterns of Intron Gain and Loss in Fungi
Source: PLoS Biol. 2004 Nov 30;2(12):e422. doi: 10.1371/journal.pbio.0020422 (PMC532390; doi:10.1371/journal.pbio.0020422)
Supplement: Table S1 — Also available at http://genes.mit.edu/NielsenEtAl/. (4.3 MB ZIP). [file pbio.0020422.st001.zip › NielsenEtAl/html/593.html]

AN1394.1.NCU03515.1.MG06726.1.FG06035.1


```
 CLUSTAL W (1.82) Multiple Sequence Alignments - Introns Inserted


Sequence 1: MG06726.1	337 aa
Sequence 2: FG06035.1	339 aa
Sequence 3: NCU03515.1	337 aa
Sequence 4: AN1394.1	342 aa
Alignment Length: 343 aa
Number Identitical Residues: 274 aa
Alignment Score (without introns) 11138


MG06726.1 	MAAMP--AATIYPQSHVGFDSITSQIERKLLKRGFQFNVICVG1QTGLGKSTLINTIFAS
NCU03515.1	MSTMG--PATVHPETHVGFDSITSQIERKLLKRGFQFNVICVG1QTGLGKSTLINTIFAS
FG06035.1 	MAAAP--PNTIYPESHVGFDSITSQIEKKLLKRGFQFNVICVG1QTGMGKSTLINTIFAS
AN1394.1  	MATTSSAPSTVFPRSHVGFDSITSQIERKLLKRGFQFNVMCVG1QTGLGKSTLINTIFAS
          	*::  ::. *:.*.:************:***********:*** ***:************

MG06726.1 	HLIETKGRHHPDEVIRSTTEIHPVSHI~IEENGVRLRLNIVDTPGYGDLINNDRC~WDPI
NCU03515.1	HLIDSKGRLQPDETVRSTTEIQSVSHI~IEENGVRLRLNIVDTPGYGDMINNDRC~WDPI
FG06035.1 	HLIDSKGRFQPDEPIRQTTEIQAVSHN~IEENGVRLRLNIVDTPGYGDLVNNDRC~WDPI
AN1394.1  	HLIDSKGRLTPNEPVRSTTEIQTVSHI1IEENGVRLRLNIVDTPGYGDQVNNDRC2WDPI
          	***::***  *:* :*.****:.***  ******************** :***** ****

MG06726.1 	VKYIKDQHSAYLRKELTAQRERYIQDTRIHCCLFFIQPSGHS~LKPIDIVVLKKLSDVVN
NCU03515.1	VKYIKDQHSAYLRKELTAQRERYIQDTRIHCCLFFIQPSGHS~LKPIDIVVLKKLSDVVN
FG06035.1 	VKYIKDQHSAYLRKELTAQRERYIQDTRIHCCLFFIQPSGHS~LKPIDIVVLKKLSDVVN
AN1394.1  	VKYIKDQHSAYLRKELTAQRDRYIQDTRIHCCLFFIQPSGHA2LKPIDIVVLKKLSDVVN
          	********************:********************: *****************

MG06726.1 	VVPVIAKADSLTLEERQAFKERIKEEFAFHNLKMYPYDNDDLDEDERSHNQQIK0A-TVP
NCU03515.1	VVPVIAKSDSLTLEERQAFKERIKEEFAFHNLKMYPYDNDELDEEERSLNQQIK0S-LIP
FG06035.1 	VVPVIAKADTLTVEERQEFKERIKEEFAFHNLKMYPYDNDEFDDEERALNGQIK0QNLVP
AN1394.1  	VVPVIAKADSLTLEERQAFKERIKEEFAFHNLKMYPYDNDELDDEERAVNARIK0D-IIP
          	*******:*:**:**** **********************::*::**: * :**    :*

MG06726.1 	FAVVGSEKSIIVNGKQVRGRQNRWGVINVEDEEHCEFVHLRNFLLRTHLQDLIETTSQIH
NCU03515.1	FAVVGSEKSIVVNGKQVRGRQNRWGVINVEDENHCEFIYLRNFLLRTHLQDLIETTSQIH
FG06035.1 	FAVVGSEKSIIVNGKQVRGRQNRWGVINVEDETHCEFVYLRDFLLRTHLQDLIETTSQIH
AN1394.1  	FAVVGSEKTIVVNGQPVRGRQNRWGVINVEDESHCEFVSLRNFLTRTHLQDLIETTSQIH
          	********:*:***: **************** ****: **:** ***************

MG06726.1 	YETFRTKQLLALKDPSAQG-HSS--RPISPAAEREMSRSSQRMTMNGY
NCU03515.1	YETFRAKQLLALKESSAQG-HSS--RPISPAADREMSRNSQRMTMNGY
FG06035.1 	YETFRAKQLLALKESSAHGGASS--RPISPAADRELSRNSQRMTMNGY
AN1394.1  	YETFRAKQLLALKESSAAGGHTSGSRPISPSADRELSRNSQRVTMNGY
          	*****:*******:.** *. :*.:*****:*:**:**.***:*****
```
